# Electrophysiological indices of pain expectation abnormalities in fibromyalgia patients

**DOI:** 10.3389/fnhum.2022.943976

**Published:** 2022-09-30

**Authors:** Paloma Barjola, Irene Peláez, David Ferrera, José Luis González-Gutiérrez, Lilian Velasco, Cecilia Peñacoba-Puente, Almudena López-López, Roberto Fernandes-Magalhaes, Francisco Mercado

**Affiliations:** Department of Psychology, Faculty of Health Sciences, Rey Juan Carlos University, Madrid, Spain

**Keywords:** pain expectations, CNV, LEPs, fibromyalgia, pain processing, P2

## Abstract

Fibromyalgia is a chronic pain syndrome characterized by dysfunctional processing of nociceptive stimulation. Neuroimaging studies have pointed out that pain-related network functioning seems to be altered in these patients. It is thought that this clinical symptomatology may be maintained or even strengthened because of an enhanced expectancy for painful stimuli or its forthcoming appearance. However, neural electrophysiological correlates associated with such attentional mechanisms have been scarcely explored. In the current study, expectancy processes of upcoming laser stimulation (painful and non-painful) and its further processing were explored by event-related potentials (ERPs). Nineteen fibromyalgia patients and twenty healthy control volunteers took part in the experiment. Behavioral measures (reaction times and subjective pain perception) were also collected. We manipulated the pain/no pain expectancy through an S1–S2 paradigm (cue-target). S1 (image: triangle or square) predicted the S2 appearance (laser stimulation: warmth or pinprick sensation). Laser stimuli were delivered using a CO_2_ laser device. Temporal and spatial principal component analyses were employed to define and quantify the ERP component reliability. Statistical analyses revealed the existence of an abnormal pattern of pain expectancy in patients with fibromyalgia. Specifically, our results showed attenuated amplitudes at posterior lCNV component in anticipation of painful stimulation that was not found in healthy participants. In contrast, although larger P2 amplitudes to painful compared to innocuous events were shown, patients did not show any amplitude change in this laser-evoked response as a function of pain predictive cues (as occurred in the healthy control group). Additionally, analyses of the subjective perception of pain and reaction time indicated that laser stimuli preceded by pain cues were rated as more painful than those signaling non-pain expectancy and were associated with faster responses. Differences between groups were not found. The present findings suggest the presence of dysfunction in pain expectation mechanisms in fibromyalgia that eventually may make it difficult for patients to correctly interpret signs that prevent pain symptoms. Furthermore, the abnormal pattern in pain expectancy displayed by fibromyalgia patients could result in ineffective pain coping strategies. Understanding the neural correlates of pain processing and its modulatory factors is crucial to identify treatments for chronic pain syndromes.

## Introduction

Chronic pain is a complex pathological state influenced by numerous physiological, psychological and social factors. Fibromyalgia, as a chronic pain syndrome, has sparked interest in the scientific field due to its unknown etiology and unclear pathophysiological mechanisms. Nevertheless, there is no doubt that psychoneurobiological dysfunctions might have crucial roles in the multifactorial symptomatology of this clinical condition (Schweinhardt et al., 2008; Lee et al., 2011; Murga et al., 2017; Richard et al., 2019).

Beyond widespread pain, patients commonly have additional physical, affective and cognitive alterations (Aparicio et al., 2013; Bell et al., 2018; Ferrera et al., 2021; Mercado et al., 2022). Indeed, current evidence has confirmed the principal role of attentional and affective processes in influencing pain hypersensitivity in fibromyalgia (Peters et al., 2000; Gracely et al., 2002; Geisser et al., 2003; Giesecke et al., 2005; Bartley et al., 2009; Duschek et al., 2014; Ellingson et al., 2018). Of note, attention processing abnormalities, mainly consisting of attentional biases or hypervigilance to pain (Schoth et al., 2012; Crombez et al., 2013; Van Ryckeghem and Crombez, 2015; Todd et al., 2018; Van Ryckeghem et al., 2019), have been reported as a contributing factor to initiate, exacerbate and perpetuate augmented pain processing in chronic pain patients as a result of the perceived threat (Vlaeyen et al., 2016). Although increasing research supports the hypothesis that patients with fibromyalgia seem to manifest a preferential allocation of attention toward pain-related stimulation (González-Roldán et al., 2013; Lai et al., 2021; Fernandes-Magalhaes et al., 2022), other studies have failed to find such attentional bias in fibromyalgia patients (Sitges et al., 2018; Pidal-Miranda et al., 2019). It has been proposed that hypervigilance to pain is a dynamic mechanism that activates whenever patients anticipate or interpret pain as a threat, promoting escape and avoidance behaviors, and it is also involved in the increase in pain perception and severity of fibromyalgia (Van Damme et al., 2015).

The expectation of pain seems to be one of the most relevant attentional factors modulating pain experience (Rainville, 2002; Ploghaus et al., 2003; Apkarian et al., 2005; Wiech et al., 2008; Knudsen et al., 2011; Wiech, 2016). Experimental studies focused on pain expectation processing have usually employed predictive cues before presenting an impending somatosensory stimulation (cue-target or S1–S2 paradigms). Applying such paradigms, it has been demonstrated that pain expectancy enhances evaluative pain-related brain regions and subsequent pain perception of painful stimuli (Sawamoto et al., 2000; Koyama et al., 2005; Keltner et al., 2006; Fairhurst et al., 2007; Moseley and Arntz, 2007; Johnston et al., 2012; Kong et al., 2013). In patients with chronic pain, including those suffering from fibromyalgia, neuroimaging studies also found enhanced activation within cerebral regions linked to evaluative pain processing, including periaqueductal gray matter, parietal, insular, cingulate, prefrontal and parahippocampal cortices, when patients were waiting for the upcoming application of a nociceptive stimulation (Cook et al., 2004; Burgmer et al., 2011; González-Roldán et al., 2016). Evidence from some electrophysiological studies has also reported a clear alteration of neural expectancy mechanisms in patients with cervical dystonia or migraine (Rizzo et al., 1985; Kaji et al., 1995), indicating reduced neural anticipatory responses. In the same way, but in contrast to the abovementioned behavioral and neuroimaging findings, fibromyalgia patients also showed decreased amplitudes of the Contingent Negative Variation (CNV) component in anticipation of upcoming pain events (Brown et al., 2014).

Event-related potentials (ERPs) represent a high temporal resolution methodology and are considered a very useful tool to reliably explore the temporal dynamics of neural networks underlying attention and emotion processes (Legrain et al., 2012; Luck, 2014; Gupta et al., 2019). Slow and late negative components of the ERPs (elicited following a predictive cue and ending just before the appearance of an impending stimulus) have been considered the neural correlates of anticipatory attentional mechanisms (i.e., CNV- or stimulus preceding negativity -SPN-; Brunia and van Boxtel, 2001; Brunia et al., 2011). Specifically, two subcomponents of CNV (early and late) have been found in association with cognitive anticipation and motor/cognitive preparation processes, respectively (Rohrbaugh and Gaillard, 1983). While early CNV (mean latency approximately 600 ms) has a mainly frontocentral scalp distribution, late CNV (starting at 800 ms from cue onset) has shown maximal amplitudes in the parieto-occipital scalp regions (Rosahl and Knight, 1995; Cui et al., 2000; Carretié et al., 2001a; Gómez et al., 2003). Thus, it is thought that enhanced amplitudes of expectation-related waves preceding a painful stimulus represent the mobilization of attentional resources to prepare the organism to effectively process and react to it as a relevant and potential threat (Brown et al., 2008; Brown, 2017).

Moreover, pain expectations have the capacity to modulate neural networks involved in pain processing along with the subsequent pain experience (Sawamoto et al., 2000; Atlas and Wager, 2012; Jepma et al., 2018; Shih et al., 2019; Henderson et al., 2020). Electrical (Hird et al., 2018), thermal (Granovsky et al., 2008), or laser painful stimulation (de Tommaso et al., 2011) have all been used as different kinds of nociceptive signals for eliciting pain-related neural correlates. In particular, laser-evoked potentials (LEPs) have been repeatedly described as good neural indices of pain processing (Treede et al., 2003; Plaghki and Mouraux, 2005). For instance, the P2 component of LEPs, with a maximal amplitude at Cz and a mean peak latency of 300–360 ms (Kakigi et al., 2004), has accumulated considerable evidence about its involvement in attentional aspects of pain processing, since its amplitude increases when individuals are paying attention to painful stimulation (Beydoun et al., 1993; García-Larrea et al., 1997; Lorenz and García-Larrea, 2003; Boyle et al., 2008). Interestingly, an increase in P2 amplitudes in response to laser stimulation has also been shown in fibromyalgia (Gibson et al., 1994; Lorenz et al., 1996; Lorenz, 1998; de Tommaso et al., 2011, 2017), suggesting that it may be considered a reliable neural index of attentional influences on pain processing in this chronic syndrome.

Different cognitive mechanisms have been proposed to modulate the P2 component (Brown et al., 2014; de Tommaso et al., 2011; Peláez et al., 2019; Vecchio et al., 2022). Some data have reported that both P2 amplitudes and subjective pain perception could be modulated by the amplitude of expectancy ERP components (e.g., early SPN) elicited by pain-informative cues (Brown et al., 2008). Moreover, the manipulation of the predictive value of pain cues may affect cortical activation; thus, incongruent cue-target associations produce greater neural responses to somatosensory stimulation than congruent ones (Dowman, 2004, 2011; Lorenz et al., 2005; Huang et al., 2017). However, such a relationship between pain anticipatory mechanisms and pain processing neural indices was not confirmed in fibromyalgia patients (Brown et al., 2014). As mentioned, to date, only a few studies have focused on the electrophysiological neural indices of pain expectancy and their potential effect on pain processing in patients with fibromyalgia.

Therefore, the present study aimed to explore the neural mechanisms of pain expectancy and their influence on the further processing of painful events in patients with fibromyalgia through the analysis of ERPs. Whereas the detection and quantification of CNV allows for the study of anticipatory attention mechanisms toward upcoming stimulation (painful or non-painful), analysis of the P2 component of LEPs provides a reliable neural correlate of pain processing. According to prior data about hypervigilance to pain, we hypothesized neural abnormalities in anticipatory responses (CNV) to painful events in fibromyalgia. This alteration in pain expectancy mechanisms might influence brain processing of pain (P2), its subjective perception, and the behavioral outcome (reaction time) in these chronic pain patients.

## Materials and methods

### Participants

Twenty-three female fibromyalgia patients and 26 female healthy control participants with an age range from 35 to 65 years old participated in this study. However, data from only 19 patients and 20 controls were considered for further analysis, as will be fully explained later. Patients were diagnosed according to the American College of Rheumatology (ACR) criteria for fibromyalgia (Wolfe et al., 1990, 2016) and recruited from the “Pain Unit” belonging to the Alcorcón Foundation-Hospital (Madrid, Spain) and the association of fibromyalgia patients of Madrid “AFIBROM.” Healthy control participants were recruited by emailing the whole university community of Rey Juan Carlos University (Madrid, Spain) and placing advertisements along the university campus. All participants were right-handed and had normal or corrected-to-normal vision. Participants with neurological (stroke, traumatic brain injury), ischemic or psychiatric disorders that might impair cognitive functioning were excluded from the study. The groups were matched by age [t(1,37) = 1.43, *p* = 0.161, *d* = 0.45] and educational level [χ2(1,4) = 5.46, *p* = 0.243, *V* = 0.37].

At the time of the study, 89% of the patients were taking medication (analgesics, antidepressants, anxiolytics, antiepileptics, or psycholeptics) to manage their clinical symptoms. Due to both ethical and medical considerations, the patients were allowed to continue taking medication, but their possible effects on their behavioral and brain electrical responses were analyzed through statistical contrasts. Only one control participant occasionally took analgesic medication on the days of the experimental protocol. The sociodemographic and psychological measures of the patients whose data were finally processed are shown in Table 1, along with the information about their medication.

**TABLE 1:** Sociodemographic and clinical measures.

	Fibromyalgia (*n* = 19)	Healthy control (*n* = 20)	*T*-test/ chi square	Significance level	Effect size
**Age (years. M ± sd)**	51.53 ± 6.12	48.40 ± 7.43	*t* = 1.43	*p* = 0.161	*d* = 0.45
**Educational level. *n* (%)**			χ^2^ = 5.46	*p* = 0.243	*V* = 0.37
Unqualified	1 (5.3%)	0 (0%)	–	–	–
Primary school	7 (36.8%)	4 (20%)	–	–	–
Secondary school	7 (36.8%)	5 (25%)	–	–	–
High school	3 (15.8%)	9 (45%)	–	–	–
Bachelor degree	1 (5.3%)	2 (10%)	–	–	–
**Symptoms duration (years. M ± sd)**	17.75 ± 8.22	–	–	–	–
**Diagnostic duration (years. M ± sd)**	7.43 ± 5.05	–	–	–	–
**Medication. *n* (%)**					
Antidepressants	12 (63.2%)	0 (0%)	–	–	–
Anxiolytics	6 (31.6%)	0 (0%)	–	–	–
Analgesics/Opioids/NSAIDs	13 (68.4%)	1 (5%)	*t* = 5.26	*p* < 0.001	*d* = 1.68
Antiepileptics/Psycholeptics	5 (26.3%)	0 (0%)	–	–	–
**Trait anxiety (STAI-T percentile. M ± sd)**	71.11 ± 31.33	30.30 ± 29.25	*t* = 4.20	*p* < 0.001	*d* = 1.34
**State anxiety (STAI-S percentile. M ± sd)**	58.63 ± 28.23	13.37 ± 12.87	*t* = 6.38	*p* < 0.001	*d* = 2.06
**Depression (BDI total score. M ± sd)**	19.50 ± 13.25	5.40 ± 5.35	*t* = 4.31	*p* < 0.001	*d* = 1.39
**Pain intensity and impact (WHYMPI-I. M ± sd)**					
Item 1–Pain intensity	3.37 ± 1.21	–	–	–	–
Pain interference	4.52 ± 1.19	–	–	–	–
Social support	4.17 ± 1.69	–	–	–	–
Pain severity	4.10 ± 0.86	–	–	–	–
Perceived control	3.78 ± 1.56	–	–	–	–
Affective distress	3.10 ± 1.51	–	–	–	–
**Responses by others (WHYMPI-II. M ± sd)**					
Negative	1.61 ± 1.08	–	–	–	–
Solicitous	2.86 ± 1.28	–	–	–	–
Distracting	3.03 ± 1.40	–	–	–	–
**Everyday activities (WHYMPI-III. M ± sd)**					
Household chores	4.58 ± 1.06	–	–	–	–
Outdoor work	1.46 ± 1.19	–	–	–	–
Activities away from home	2.67 ± 1.31	–	–	–	–
Social activities	2.52 ± 1.21	–	–	–	–
**WHYMPI general activity score (M ± sd)**	2.83 ± 0.92	–	–	–	–

Mean and standard deviation (M ± sd), statistics of the group comparisons (Student’s t-test or χ2-tests), *p*-value and effect sizes (if applicable) are reported for fibromyalgia and healthy control groups (for the final sample size, see above).

(NSAID, non-steroidal anti-inflammatory drugs; STAI, State Trait Anxiety Inventory; BDI, Beck Depression Inventory; WHYMPI, West-Haven Yale Multidimensional Pain Inventory).

Participants gave written informed consent for their involvement in the experiment. The Rey Juan Carlos University Research Ethics Board approved this study according to the ethical principles for research conducted with human participants required by this committee. Just before starting the experimental session, different self-report instruments were administered to the participants to characterize their emotional and physical health conditions. The affective status of the participants was assessed by application of the Beck Depression Inventory (Beck et al., 1961) and the State-Trait Anxiety Inventory (Spielberger et al., 1982). Fibromyalgia patients had higher anxiety and depression scores than healthy control participants (see Table 1). Additionally, the patients were asked to provide information about the time elapsed since the onset of symptoms and their diagnosis. They also completed the West Haven-Yale Multidimensional Pain Inventory (Kerns et al., 1985) to assess several characteristics of the experienced chronic pain (Table 1).

### Stimuli and procedure

**FIGURE 1:**
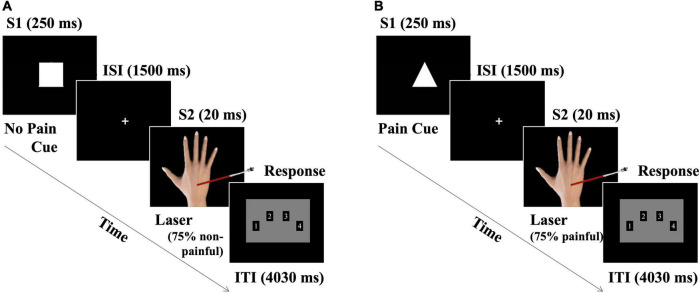
Schematic representation of the experimental procedure. The S1–S2 paradigm is displayed, where S1 = cue (visual) correctly predicted the intensity of the upcoming S2 = target (laser stimulation) in 75% of the trials. **(A)** No Pain cue trial, and **(B)** pain cue trial representations. The interstimulus interval (ISI) was 1,500 ms. Intertrial interval (ITI) = 4,030 ms. Only responses within the first 2,000 ms of ITI were considered.

An S1–S2 (cue–target) paradigm was used to manipulate the pain expectations and analyze their influence on brain electrical responses to laser stimulation (see Figure 1). The subjective perception of laser stimulation was also measured. Thus, two types of stimuli were presented: a picture representing a geometric figure (S1: white triangle or square) followed by an upcoming laser stimulus (S2: painful or non-painful). Each S1 informed about the nature of the subsequent laser stimulation, configuring two types of cues (Pain cue -PC- or No Pain cue -NPC-). The visual cue stimuli (S1) were matched in size (5 cm height × 5 cm width). They were presented for 250 ms at the center of a 19′′ screen against a black background. Each of them predicted the appearance of a painful (P) or non-painful (NP) laser stimulation. The correspondence between them (S1 and S2) was counterbalanced (i.e., triangle-PC and square-NPC for 50% of the participants).

As the target stimulation (S2), brief laser pulses were delivered over the dorsum of the participants’ dominant hand through a CO_2_ laser (Neurolas, Electronic Engineering; wavelength of 10.6 μm) with a power of 6 watts. Two different laser intensities were set at infra- and supra-threshold pain levels: 15.3 mJ/mm^2^ (beam diameter of 2.5 mm) for eliciting a “*pinprick*” or painful sensation (P) and 10.9 mJ/mm^2^ (beam diameter of 5 mm) for a “*warmth*” or non-painful sensation (NP). The modification of the laser pulse intensities was made *via* the beam diameter; the smaller the beam diameter was, the more painful the stimuli. Participants were asked to inform, as soon as possible, about the perceived intensity associated with S2.

Before the experimental session, the two thresholds were determined using the method of limits. Participants experienced 6 ascending and descending series of 10 laser stimuli using the method of limits, as recommended in prior studies (Weiss et al., 2003; Peláez et al., 2016). Participants were asked to verbally inform about the perception of the stimuli (no sensation–sensation) and the intensity of the perceived stimuli (warmth or painful). All participants described the 5 mm diameter laser stimulation as an innocuous warmth sensation, while the 2,5 mm one was established as a tolerable painful pinprick sensation. To avoid tissue damage, habituation or nociceptor sensitization, the laser stimulation was shifted approximately 2 cm after each trial.

The stimuli sequence was controlled by the Gentask module of the STIM2 software (Compumedics Neuroscan). Participants and researchers wore protective goggles during all phases of the experimental procedure. After each laser stimulus, participants were instructed to report the perceived intensity of the laser stimulation through a four-button response pad (1 = “no pain,” 2 = “low pain,” 3 = “moderate pain,” and 4 = “extreme pain”). To prevent impulsive responses, a white dot was displayed on the screen simultaneously with the appearance of a laser stimulus, indicating to the participants the moment for giving their responses. Any response given above 2,000 ms was identified and removed from further analyses.

To explore the pain expectation influences on pain processing, the cue probability for correctly informing about the nature of the subsequent impending laser stimulation (P or NP) was manipulated. Thus, each kind of cue only correctly predicted each type of laser stimulation for 75% of the trials. This manipulation led to four experimental conditions: (1) PC-P, (2) PC-NP, (3) NPC-NP, and (4) NPC-P, among which two congruent (cues correctly predicted laser intensity) and two incongruent (cues incorrectly predicted laser intensity) conditions were established. Participants were told that the cue stimuli correctly predicted the intensity of the laser stimulation in most trials, but some trials contained invalid pain cues. The experimental session included a total of 320 pseudorandomized trials (160 PC/P trials and 160 NPC/NP; 50% each). Among them, 120 trials were congruent (75%), and 40 trials were incongruent (25%). The same experimental condition was not displayed in more than three consecutive trials. The total stimulation was distributed in 8 blocks of 40 trials, maintaining the mentioned proportions of congruent and incongruent trials. After each block, the participants were offered an optional short break (1–2 min) to minimize fatigue. Each trial lasted 5,800 ms with a fixed intertrial interval (ITI) of 4,030 ms (the experimental paradigm can be seen in Figure 1). The entire experimental paradigm lasted 32 min (4 min per block). Before the experimental procedure, the participants completed at least two practice sequences of ten trials to ensure their understanding of the task.

### Electroencephalography recording and pre-processing

Participants were seated in a light- and sound-attenuated and electrically isolated room, facing a screen placed 90 cm away from their face. Electroencephalographic (EEG) activity was recorded using a cap with 60 electrodes homogeneously distributed over the scalp (QuickCap Neuroscan). In addition, vertical and horizontal electrooculographic (EOG) activity was recorded from two electrodes located supra- and infra-orbitally on the left eye and two electrodes located on the right and left orbital rims. All electrodes were referenced to the mastoids, and impedances were kept below 5 kΩ. During the entire recording session, channels continuously digitized the data at a sampling rate of 250 Hz. An online bandpass filter was set from 0.1 to 50 Hz. A notch filter at 50 Hz was also applied. Offline pre-processing was performed using Brain Vision Analyzer software v.1.0 (Brain products). Data were digitally filtered using a 30 Hz low pass filter. Segmentation of the continuous recording was carried out on the two different time windows regarding the laser stimulation: the *Expectation* period (after S1 and before S2) and the *Pain processing* period (after S2). The expectation period was divided into 1,700 ms epochs for each trial, beginning 200 ms before S1 onset, and two experimental conditions were defined (PC and NPC). Epochs of 1,000 ms (−200 to 800 ms, from S2 onset) were selected for the pain processing period, where four experimental conditions were established (PC-P, PC-NP, NPC-NP, and NPC-P). The EOG-artifact removal procedure was carried out according to the procedure described by Gratton et al. (1983). The artifact detection threshold was set at ± 70 μV. After that, baseline correction and EEG visual inspection were also carried out to remove epochs with artifacts (in both the expectation and pain processing periods) for further analyses. This artifact rejection procedure led to an average epoch admission of 60.3% PC, 59.3% NPC, 73.2% PC-P, 75.3% PC-NP, 73.5% NPC-NP, and 69.6% PC-NP trials. ERP averages were categorized according to each type of stimulus (2 types of cues for the expectation period and 2 cues × 2 laser intensities for the pain processing period). Three participants (2 healthy controls and 1 fibromyalgia) were excluded from the study due to the lack of perception of innocuous stimuli during the discrimination series conducted prior to the experimental session. Furthermore, data from 4 healthy controls and 3 fibromyalgia participants were excluded from further analyses because of the high rate of artifacts in the EEG recording (over 50% epochs rejected). Finally, data from 39 participants (19 fibromyalgia patients and 20 healthy controls) were analyzed.

### Statistical analyses

#### Detection and quantification of event-related potentials

Temporal principal component analysis (tPCA) using a covariance matrix was employed to reliably detect and define the ERP components explaining most of the brain electrical activity variance in response to the predictive cues (expectation period) and laser stimulation (pain processing period). This statistical method for dimension reduction has been strongly recommended when high-density montages are employed, since it avoids subjectivity and misinterpretation in the selection of the time windows based on visual inspection of the grand-averaged ERPs. Thus, the resulting ERP components are free of the influences of other adjacent or subjacent components [see, Dien and Santuzzi, 2005)] for a more detailed description of the tPCA procedure and advantages]. This technique has already demonstrated its ability to disentangle and characterize ERP components, and it has been previously used for the study of expectation (i.e., CNV; Carretié et al., 2001b, 2004) and LEPs (Peláez et al., 2019). In brief, tPCA computes the covariance between all ERP time points, which tends to be high between those time points involved in the same component and low between those belonging to different components. The solution provides a set of different temporal factors made up of highly covarying time points, each of them theoretically involved in the same ERP component. The temporal factor (TF) score, the tPCA-derived parameter in which the extracted TFs may be quantified, is linearly related to the amplitude value of the ERP components. In the present study, thirty-nine subjects, two stimulus categories (PC and NPC) and sixty electrode sites yielded a total of 4,680 averaged waveforms, which served as the database for the tPCA related to the expectation period. In addition, thirty-nine subjects, four stimulus categories (PC-P, PC-NP, NPC-NP, and NPC-P) and sixty electrode sites yielded a total of 9,360 averaged waveforms, which served as the database for the tPCA related to the pain processing period.

Considering that signal overlapping may also occur in the space domain, a spatial principal component analysis (sPCA) was applied. This method allows us to reliably define the topographical distribution of ERP components and decompose the TFs into their main spatial regions or spatial factors (SFs) by means of the detection of covarying scalp points. Likewise, different neural processes may concur at any given time point, corresponding to different electrical signals, and the recording at any scalp location at that moment represents the electrical balance of these distinct neural processes. While tPCA allows for representing the complex superposition of different overlapping ERP components in time, sPCA separates the ERP components along the electrodes located on the scalp, providing a set of SFs of highly covarying scalp regions. These resulting SFs ideally may represent the concurrent neural processes underlying each TF. This configuring and quantifying scalp regions system is preferable to an *a priori* subdivision into fixed scalp regions for ERP components, since sPCA demarcates scalp regions according to the real behavior of each scalp-point recording. The number of extracted factors in both the temporal and spatial PCA was determined by the scree test method (Cattell, 1966). Promax rotation was applied to select both temporal and spatial factors (Dien, 2010, 2012).

#### Experimental effects on event-related potentials and behavior

To explore the pain expectations and their influence on pain processing at both the neural and behavioral levels, a series of repeated-measures ANOVAs was computed, as described below:

1. Neural response to pain predictive cues (expectation period): 2 × 2 repeated-measures ANOVAs, where cue (PC and NPC) was introduced as a within-subject factor and group (fibromyalgia and healthy control) was used as a between-subjects factor, were carried out to explore the effects on the CNV component (early and late) of the ERPs.
2. Neural response to laser stimulation (pain processing period): 2 × 2 × 2 repeated-measures ANOVAs were computed to explore the effects of pain expectations on the P2 LEP component. Here, the selected within-subject factors were cue (PC and NPC) and type of laser stimulus (P and NP). The group of participants (fibromyalgia and healthy control) was entered as the between-subjects factor.
3. Behavioral measures: Reaction time and perceived pain intensity in response to laser stimulation were analyzed by a series of 2 × 2 × 2 repeated-measures ANOVAs, including the same factors previously described in the pain processing period for testing the effects of pain expectations on these two variables.

To determine the significance and direction of the mentioned contrasts, *post-hoc* comparisons using the Bonferroni’s test were carried out (alpha = 0.05). Greenhouse− Geisser (GG) epsilon correction was applied to adjust the degrees of freedom of the F statistic. Effect sizes were computed through the eta-square (η^2^_*p*_) method. Finally, with the aim of detecting potential influences of medications on attentional and pain-related processes, repeated-measures ANOVAs (Greenhouse−Geisser correction) were also computed, using the consumption of drugs as between-subjects factors and the ERP components of interest (i.e., CNV and P2) and behavioral measures as within-subject factors. All statistical analyses were performed using SPSS Statistics 25 (IBM, Inc.).

## Results

### Event-related potential data

#### Pain expectation period

**FIGURE 2:**
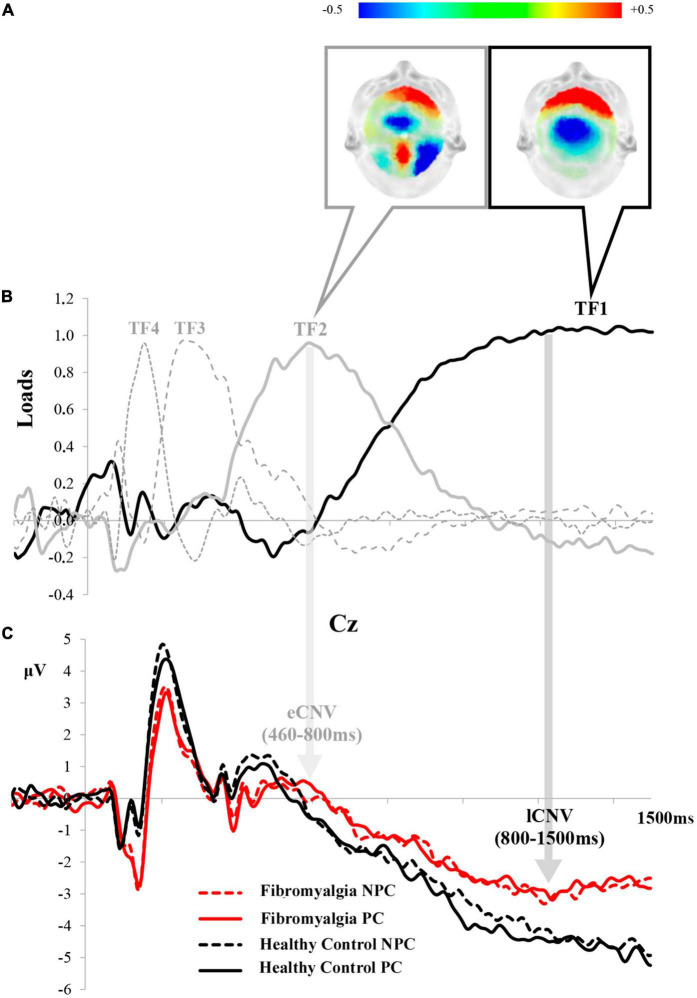
Correspondence between TFs and ERP components derived from the tPCA in the expectation period: **(A)** topographical distribution of early (TF2) and late (TF1) CNV; **(B)** TF loads after the application of tPCA; and **(C)** the correspondence of each component in the grand average of Cz. (PC, pain cue; NPC, no pain cue).

The tPCA application on the interval between S1 and S2 led to the extraction of four TFs. Considering both their latency (starting at 800 and 460 ms) and topographic distribution (showing a maximal amplitude at the parieto-occipital and frontal scalp sites), TF1 and TF2 were associated with the ERP components signaled in the grand averages as lCNV and eCNV, respectively. Figure 2 shows the correspondence between the TFs derived from the tPCA and the ERP components in the expectation period. Through the subsequent application of the sPCAs to TF scores, two SFs or scalp regions were established for lCNV: SF1 (the posterior scalp region) and SF2 (the frontal scalp region). The whole TF corresponding to the eCNV component was divided into four SFs or scalp regions: SF1 (the frontal scalp region), SF2 (the posterior scalp region), SF3 (the central scalp region), and SF4 (the left temporal-parietal scalp region).

**FIGURE 3:**
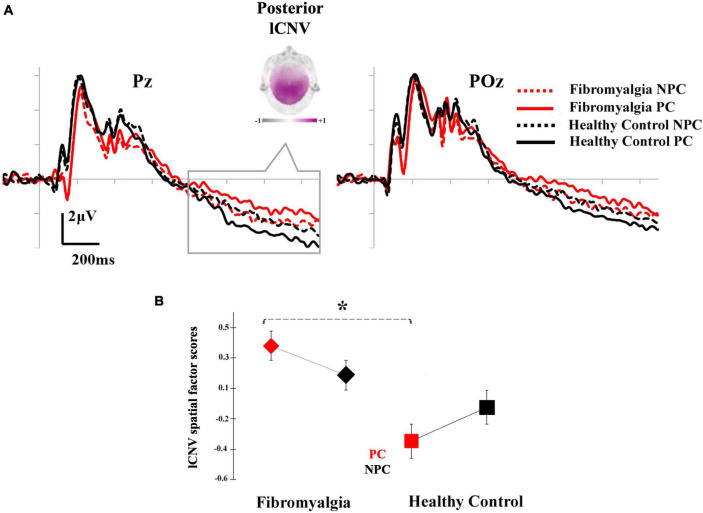
Grand averages of the expectation period: **(A)** ERP waveforms for fibromyalgia patients (red) and healthy control participants (black) in response to PC (solid lines) and NPC (dashed lines) at the posterior selected electrodes (Pz and POz). The time window (800–1,500 ms, gray rectangle) and topographic map of lCNV are also highlighted; and **(B)** means of the lCNV spatial factor scores (posterior region) for fibromyalgia (left) and healthy control participants (right) in PC (red) and NPC (gray) conditions. Asterisks show the comparison in which significant differences were found. Bars show the standard error (*p < 0.05). (PC, pain cue; NPC, no pain cue).

Repeated measures ANOVAs showed significant differences in the posterior lCNV component for the interaction between Cue × Group [*F*_(1,37)_ = 5.580, *p* = 0.024, η^2^_*p*_ = 0.131]. *Post-hoc* comparisons revealed a lower amplitude of posterior lCNV in fibromyalgia patients than in healthy control participants after the pain prediction cues. Figure 3 displays the grand averages of the posterior lCNV, where the experimental effects can be observed. Other effects associated with Group or Cue factors did not reach statistical significance. Analyses conducted on the eCNV revealed that this ERP component was not sensitive to experimental manipulations related to pain expectation (*p* > 0.05 for all of the contrasts).

#### Pain-processing period

**FIGURE 4:**
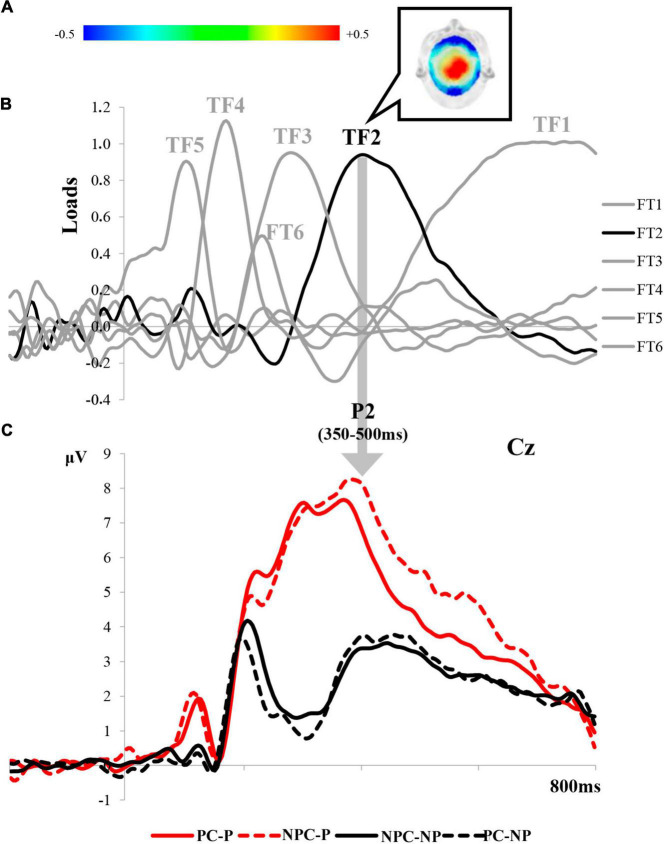
Correspondence between TFs and ERP components derived from the tPCA in the pain-processing period: **(A)** topographical distribution of the P2 of the LEPs; **(B)** TF loads after the application of tPCA; and **(C)** correspondence of each component of the LEPs in the grand average of Cz. (PC-P, pain cue - pain; NPC-P, no pain cue - pain; NPC-NP, no pain cue - no pain; PC-NP, pain cue - no pain).

Grand averages of the electrical brain activity in response to laser stimulation and the correspondence between the TFs and LEP components for the pain-processing period are displayed in Figure 4. After tPCA application in this time window, six TFs were extracted. Among them, latency (approximately 400 ms) and topographical features of TF2 (maximal amplitude at the central scalp sites in response to painful laser stimuli) corresponded with the P2 LEP waveform. Three SFs or scalp regions were extracted from TF2 (P2 component) after the application of the sPCA (see Figure 4): SF1 (the posterior scalp region), SF2 (the frontal scalp region) and SF3 (the central scalp region).

**FIGURE 5:**
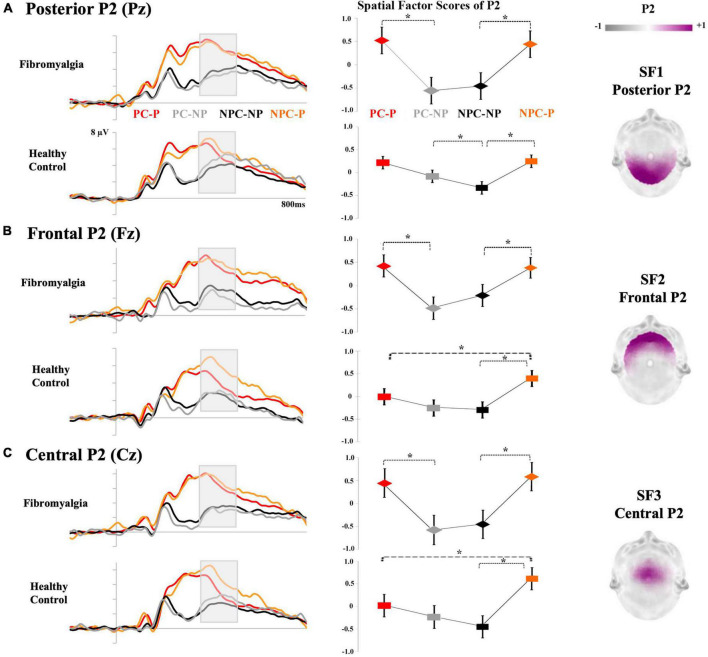
Grand averages of LEP components (pain-processing period): The left side of the figure represents the P2 waveform for fibromyalgia and healthy control groups in response to PC-P (red), NPC-P (orange), PC-NP (gray) and NPC-NP (black) conditions. The means of the P2 spatial factor scores for each experimental condition are displayed in the middle. Asterisks represent significant differences (*p < 0.05). Bars indicate the standard error. The topographical maps of each scalp region of P2 are shown on the right side. **(A)** Pz for posterior P2, **(B)** Fz for frontal P2, and **(C)** Cz for the central P2 scalp regions. (PC-P, pain cue - pain; NPC-P, no pain cue - pain; NPC-NP, no pain cue - no pain; PC-NP, pain cue - no pain).

Repeated measures ANOVAs on P2 revealed a significant main effect of laser stimulation in all the scalp regions: posterior [*F*_(1,37)_ = 36.903, *p* < 0.001, η^2^_*p*_ = 0.499], frontal [*F*_(1,37)_ = 23.181, *p* < 0.001, η^2^_*p*_ = 0.385], and central P2 [*F*_(1,37)_ = 36.088, *p* < 0.001, η^2^_*p*_ = 0.494]. *Post-hoc* comparisons showed larger amplitudes for painful than non-painful laser stimulation. Interestingly, a significant interaction effect between Group × Cue × Laser stimulation was also found for all of the scalp regions of P2: posterior [*F*_(1,37)_ = 4.771, *p* = 0.035, η^2^_*p*_ = 0.114], frontal [*F*_(1,37)_ = 4.696, *p* = 0.037, η^2^p 0.113], and central P2 [*F*_(1,37)_ = 7.828, *p* = 0.008, η^2^_*p*_ = 0.175]. *Post-hoc* comparisons revealed different pain expectancy influences on the neural response to the laser stimulation in each group of participants. Whereas both fibromyalgia patients and healthy control groups showed larger P2 amplitudes after painful than innocuous laser stimulation, the P2 wave did not reveal changes associated with the meaning conveyed by predictive cues in the fibromyalgia group. Only healthy control participants exhibited an electrophysiological response to laser stimulation modulated by the congruency between predictive cues (pain or no pain) and targets (painful or innocuous). Thus, incongruent conditions (PC-NP and NPC-P) elicited higher P2 amplitudes than congruent conditions. Specifically, the P2 amplitude at posterior scalp regions was greater for NP laser stimulation preceded by PC than NPC (PC-NP > NPC-NP). Both frontal and central P2 scalp regions showed greater amplitudes when the P laser stimulation was preceded by NPC compared to PC (NPC-P > PC-P). Taken together, these results suggest the presence of abnormal processing of pain predictive cues in fibromyalgia patients, who seem to be insensitive to the influences of pain expectations on P2, as evidenced by the group of healthy participants. A representation of the effects described on the P2 LEP component is displayed in Figure 5. No other significant differences involving the variables of interest were found.

### Behavioral data

**FIGURE 6:**
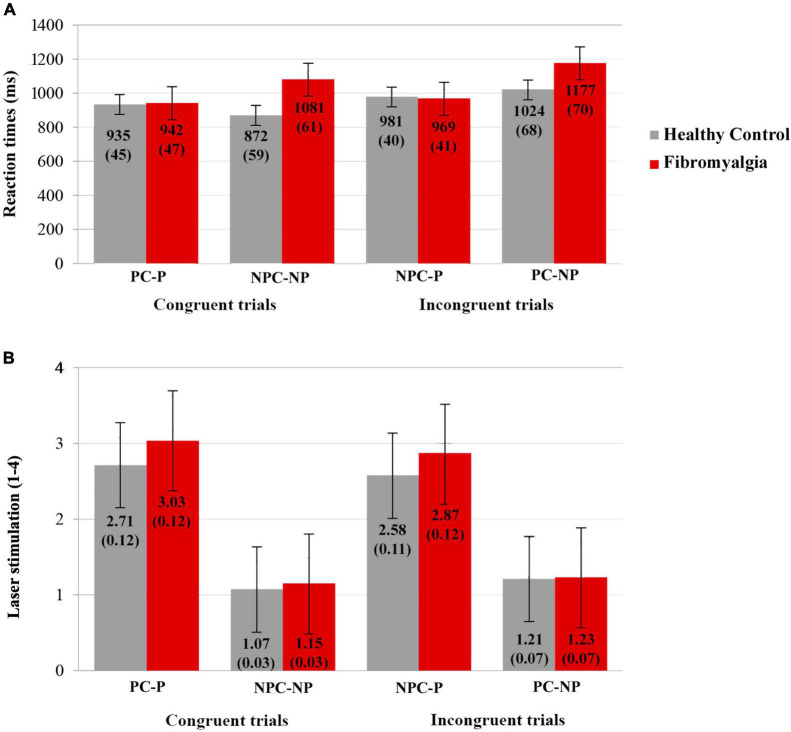
Behavioral data: Mean and standard deviation of **(A)** RTs (ms) and **(B)** subjective perception of pain (1–4) for healthy control (gray) and fibromyalgia (red) of the four experimental conditions (PC-P, pain cue-pain; NPC-NP, no pain cue-no pain; NPC-P, no pain cue-pain; PC-NP, pain cue-no pain). Bars represent the standard error.

The mean and standard deviations of the pain perception and reaction times (RTs) for each type of laser stimulus (as a function of predictive cues) are displayed in Figure 6. As expected, repeated-measures ANOVAs on subjective pain perception showed a significant main effect for laser stimulation [*F*_(1,37)_ = 340.516, *p* < 0.001, η^2^_p_ = 0.902]. *Post-hoc* analyses revealed a greater intensity of pain perception for painful (2.8 ± 0.08) compared to non-painful stimulation (1.2 ± 0.03). Additionally, a significant main effect was detected for the type of Cue [*F*_(1,37)_ = 12.766, *p* = 0.001, η^2^_p_ = 0.257]. Laser stimulation preceded by pain cues elicited a greater pain perception (2.0 ± 0.05) than no pain cues (1.9 ± 0.04). Although patients with fibromyalgia showed a higher pain perception than healthy participants, this difference did not reach statistical significance [*F*_(1,37)_ = 3.573, *p* = 0.067, η^2^_p_ = 0.088]. No other significant results involving potential interaction effects between cues and laser stimulation were found.

With respect to RTs, the results pointed out the existence of a main significant effect for type of Cue [*F*_(1,37)_ = 10.888, *p* = 0.002, η^2^_p_ = 0.227]. Longer RTs were obtained in response to pain cue trials (1020 ± 35) compared to those informing of no pain expectancy (976 ± 31). A main effect on laser stimulation was also found [*F*_(1,37)_ = 4.765, *p* = 0.035, η^2^_p_ = 0.114], according to which painful laser stimuli were associated with shorter RTs (957 ± 29) than non-painful events (1038 ± 44). In addition, a significant interaction between Cue × Laser stimulation [*F*_(1,37)_ = 23.927, *p* < 0.001, η^2^_p_ = 0.393] showed augmented RTs for incongruent trials in all of the contrasts, except for the no pain cues conditions (NP-P 975 ± 29 > P-P 939 ± 32, P-NP 1101 ± 49 > NP-NP 976 ± 42, P-NP 1101 ± 49 > P-P 939 ± 32 and NP-P 975 ± 29 = NP-NP 976 ± 42). Finally, a Group × Laser stimulation interaction was also detected [*F*_(1,37)_ = 5.980, *p* = 0.019, η^2^_p_ = 0.139]. Patients with fibromyalgia had shorter RTs for painful (956 ± 42) compared to non-painful laser stimulation (1129 ± 63). Moreover, patients were slower to inform themselves about the presence of a non-painful stimulation compared to healthy participants (948 ± 62).

#### Analysis controlling the potential effect of medication

Since 89% of patients with fibromyalgia were taking drugs by physician prescription and such medication could have effects on central nervous system (CNS) functioning, repeated-measures ANOVAs were computed to detect such possible influences in the patient group. Antidepressants and anxiolytics were introduced as between-subjects factors (due to their potential capacity to alter cognitive functioning), whereas the neural response and behavioral outcomes were defined as dependent variables. A significant effect of antidepressants was found for laser stimulation on frontal P2 amplitudes [*F*_(1,17)_ = 6.812, *p* = 0.018, η^2^_p_ = 0.286]. The P2 amplitude was higher in response to painful stimulation for patients taking antidepressant drugs, but it does not seem to add information to the present results since significant differences between groups on P2 components were not detected. lCNV amplitudes were not significantly affected by medication taken by patients with fibromyalgia.

Additionally, the impact of drug consumption on behavioral measures was also explored. A significant effect of anxiolytics for laser stimulation in RTs [*F*_(1,17)_ = 6.366, *p* = 0.022, η^2^_p_ = 0.272] was found. Thus, lower RTs were associated with painful stimulus responses in patients not taking anxiolytic medication compared to those taking them. Finally, a significant effect of antidepressants for Cue on perceived pain [*F*(_1,17)_ = 11.067, *p* = 0.004, η^2^_p_ = 0.394] was also detected. In this regard, perceived pain intensity was lower in PC trials for patients who were taking antidepressants. Again, such effects might not refute the previous results regarding RTs and perceived pain intensities.

## Discussion

The main aim of the present study was to explore the neural mechanisms of pain expectancy and its potential influence on the processing of painful and innocuous laser stimulation in fibromyalgia patients. The results could be summarized as follows: (1) patients with fibromyalgia exhibited decreased posterior lCNV amplitudes in anticipation of painful stimulation; (2) in contrast with findings about expectancy-related modulations detected in healthy participants, the P2 component was not sensitive to the expectations of pain in fibromyalgia, varying only in accordance with the intensity of the laser stimulation; and (3) behavioral measures (pain perception and RT) were, however, modulated as a function of pain anticipation signals in the whole sample of participants, so that pain-related cues were associated with higher pain perception and slower RTs than no pain cues. Implications derived from the present findings will be addressed in more detail below.

Contingent Negative Variation has been reported as an ERP component that represents anticipatory attention, comprising cognitive and motor preparation processes, before an impending event (Brunia et al., 2011). Several studies have established that the prediction of upcoming nociceptive stimuli involves the recruitment of attentional mechanisms for pain regulation (Van Damme et al., 2010; Legrain et al., 2012; Palermo et al., 2015). Thus, pain expectation would enhance the attentional resources directed to the upcoming painful event being reflected as higher amplitudes in anticipatory ERP deflections, such as CNV or analogous components (Stude et al., 2003; Babiloni et al., 2004; Brown et al., 2008; Seidel et al., 2015). According to theoretical proposals on hypervigilance to pain (Crombez et al., 2013), it would be expected that increased pain anticipation in chronic pain patients might lead to amplified pain processing responses. In this vein, several findings have highlighted the presence of augmented activation of pain processing brain regions during pain anticipation in patients with fibromyalgia (Cook et al., 2004; Crombez et al., 2004; Burgmer et al., 2011). However, in this study, fibromyalgia patients showed a pronounced attenuation of lCNV amplitudes at posterior scalp regions for trials involving pain expectations. In support of the current results, reduced frontoparietal and ventral tegmental activation after predictive pain cues has also been reported (Loggia et al., 2014). The observed discrepancies among different sources of experimental evidence could be derived from the variability in methodological and experimental procedures regarding the manipulations on pain expectancy (i.e., in Loggia et al., 2014, pain cues informed about the pain stimulation onset, while in Burgmer et al., 2011, the uncertainty of pain predictive cues was manipulated). On the other hand, electrophysiological measures would entail more suitable techniques than other neuroimaging methodologies to reliably detect the temporal dynamics of anticipatory attention to pain processing.

The absence of an enhanced lCNV elicited by predictive pain cues in patients with fibromyalgia makes a straightforward interpretation difficult and deserves further consideration. The scarce experimental ERP evidence of pain expectative mechanisms in chronic pain patients suggests a decreased CNV in anticipation of pain events (Kaji et al., 1995) or during a pain episode (Rizzo et al., 1985). More recently, lower amplitudes of a late expectation wave have been found in fibromyalgia in anticipation of moderate intensity laser stimuli compared to healthy people and osteoarthritis patients (Brown et al., 2014). Other similar data indicating CNV alterations during pain expectation or hyperalgesia conditioning paradigms have been related to associative learning dysfunction in chronic pain patients (Schneider et al., 2004; Zhang et al., 2019). Similarly, dysfunctions of associative learning mechanisms have also been described in fibromyalgia (Jenewein et al., 2013; Meulders et al., 2015; Sandström et al., 2020). Learning from past pain experiences may allow us to adjust our behavior and respond adaptively to new pain events. Therefore, deficiencies in associative learning could become the context unstable and unpredictable, making the anticipation of pain for adequate coping very difficult, and could play an important role in the development and maintenance of chronic pain syndromes (Jepma et al., 2018; Zhang et al., 2019). In accordance with that, the abnormal pain anticipation processing in our results would suggest a disability in fibromyalgia patients, preventing them from taking advantage of the contextual cues that indicate pain increase or pain relief. Moreover, considering that psychophysiological signs of motor and cognitive preparation (such as those represented by CNV) would reflect the effort in the deployment of cognitive regulation mechanisms (Jennings and van der Molen, 2005), it could be proposed that the decreased CNV in fibromyalgia might imply difficulty mobilizing attentional resources, cognitive regulation and coping processes to adequately deal with painful events.

Regarding the neural findings for the processing of painful stimulation, our results showed that the P2 amplitude was sensitive to the intensity of the CO_2_ laser stimulation, since painful stimuli generated larger P2 waves than innocuous stimuli. Furthermore, a distinguishable modulation pattern of P2 was found by predictive cues for each group of participants. In this sense, the pain expectancy manipulation led to P2 amplitude changes only for the healthy control group. Surprisingly, these variations in P2 were not found in fibromyalgia and were influenced only by the laser stimulation intensities (larger amplitudes for painful compared to non-painful conditions). Greater amplitudes of the P2 component for painful stimuli have been repeatedly reported in the general population (Beydoun et al., 1993; García-Larrea et al., 1997; Ohara et al., 2004; Iannetti et al., 2005, 2008; Dowman, 2007). Likewise, hypersensitivity in fibromyalgia, along with increased LEPs components to painful laser CO_2_ stimuli (Gibson et al., 1994; Lorenz, 1998; de Tommaso et al., 2011, 2017), have been consistently described. Nevertheless, the absence of significant differences between groups for the amplitude of P2 in the current results goes against the mentioned neural correlates of the augmented pain processing response in fibromyalgia. Brown et al. also failed to find significant differences in P2 among fibromyalgia, osteoarthritis and healthy control participants when predictive cues were used for signaling the onset of an upcoming pain stimulus. It is well established that pain expectation cues enhance the P2 component of LEPs (Miyazaki et al., 1994; Hauck et al., 2007; Brown et al., 2008). However, when the predictive value of the cues is manipulated, the effects of pain expectancy on P2 become controversial (Dowman, 2004, 2011; Lorenz et al., 2005). The lack of pain cue effects on the P2 amplitude in the fibromyalgia group appears to be consistent with some previous evidence. Similarly, cognitive and emotional manipulations have failed to modulate the brain response after nociceptive stimulation in fibromyalgia (Kamping et al., 2013; Loggia et al., 2014), indicating the existence of an endogenous pain modulation disturbance. From this point of view, decrements of neural correlates of pain expectation in fibromyalgia have been associated with an alteration of descending pain modulation mechanisms (de Souza et al., 2009; Goffaux et al., 2009; Burgmer et al., 2011; Fields, 2018; Ioachim et al., 2022), which could be related to the absence of expectative modulation of the neural response to pain.

Complementarily, behavioral outcomes involving subjective perceptions of pain and RTs were sensitive to the experimental manipulations. Regarding pain perception, the present results support the well-established ideas about their modulation as a consequence of the anticipatory processes (Moseley and Arntz, 2007; McGowan et al., 2009; Johnston et al., 2012). All of the participants reported greater pain perception during the pain cue trials. On the other hand, both pain cueing and incongruent trials led to longer RTs than no pain cueing and congruent trials. Longer RTs to pain cue incongruent trials (PC-NP) were especially remarkable among the patients, but the lack of significant differences in the interaction involving group, type of cue and laser stimulation prevents any clear interpretation. It could be suggested that attention disengagement difficulties from pain-related cues might at least partially explain the present findings (Van Damme et al., 2002, 2004; Fernandes-Magalhaes et al., 2022). According to previous data, incongruent trials might produce an interference effect (i.e., longer RTs) in the processing of somatosensory events and, thereby, might affect behavioral performance (Yiend and Mathews, 2001). Such an effect would be more interfering for patients presenting with pain attentional biases, as usually occurs in fibromyalgia (Broadbent et al., 2021). It should be noted that the behavioral outcomes and P2 amplitudes in fibromyalgia seem to be contradictory, as the RT and perceived pain intensity were modulated by pain cues and the P2 waves were not. The ERP technique allows us to reliably disentangle the temporal course of rapid and overlapped cognitive processes, and behavior outcomes are the final output. The present investigation focused on two specific ERP components (CNV and P2), but other ERP waves (in turn, other cognitive processes) could be sensitive to pain expectative manipulations, affecting behavioral measures.

Although the present investigation is one of the few to explore pain expectancy in fibromyalgia and its further influence on pain processing, several limitations should be considered. First, we applied two different (painful and innocuous) fixed (equal for all participants) laser stimulus intensities, but it is more common to individually determine the intensity of the applied painful stimuli due to the subjectivity and variability in the pain experience (Peláez et al., 2019). Nonetheless, both methods have been employed in fibromyalgia patients, and similar results have been obtained (i.e., de Tommaso et al., 2011; Brown et al., 2014). Even though our results did not indicate significant differences in perceived pain intensity between groups, we should not omit the possible influence of individual variability in the processing of painful stimulation. In addition, the four-button device used for collecting behavioral responses (perceived pain intensity and RT) may not be optimal to show the great variability in pain reports. Mainly regarding the non-painful stimulation, a succession of warmth sensations was not reflected in the rating scale (1 = no pain), while it was reproduced in the pain sensations scale (2 = low pain, 3 = moderate pain, 4 = extreme pain). Although this pain rating procedure is unable to achieve fine somatosensory discrimination, the experimental manipulations seemed to have been effective. Second, the medication taken by fibromyalgia patients had an effect on both the brain electrical response and the behavioral performance levels. Previous scientific evidence has demonstrated that this kind of drug does not affect cognition in fibromyalgia or chronic pain patients (Chapman et al., 2002; Mohs et al., 2012; Reyes del Paso et al., 2012). Nevertheless, it should be considered with caution due to its influence on pain perception.

To conclude, the present findings point out a dysfunction of the pain expectation mechanisms in fibromyalgia, probably concerning associative learning difficulties, which would lead to abnormal processing of painful stimulation that eventually could result in ineffective strategies for pain regulation. It has been suggested that a decrease in neural expectation resources underlies such unsuccessful pain coping efforts in these patients (Brown et al., 2014). Understanding neural correlates of pain processing and pain modulation factors is indispensable to achieve a better knowledge of chronic pain syndromes. In addition, the influence of current spontaneous and endogenous pain in chronic pain sufferers should be taken into account as a factor modulating pain processing. The present research represents a contribution to broadening the knowledge of pain expectation neural mechanisms and their influence on pain processing in fibromyalgia. Future research in this field would help design more effective coping strategies to improve the quality of life of chronic pain patients.

## Data availability statement

The datasets presented in this study can be found in online repositories. The names of the repository/repositories and accession number(s) can be found below: Open Science Forum https://osf.io/bhjnc/.

## Ethics statement

The studies involving human participants were reviewed and approved by the Research Ethics Committee of the Rey Juan Carlos University. The patients/participants provided their written informed consent to participate in this study.

## Author contributions

PB and FM: conceptualization, software, and writing—original draft. PB, JG-G, CP-P, AL-L, LV, and FM: data curation. PB, RF-M, and FM: formal analysis. FM: funding acquisition, project administration, and resources. PB, JG-G, LV, IP, and FM: investigation. PB, LV, JG-G, and FM: methodology. CP-P and FM: supervision. PB, IP, DF, AL-L, RF-M, and FM: validation. PB: visualization. PB, IP, DF, RF-M, and FM: writing—review and editing. All authors have read and agreed to the published version of the manuscript.

## References

[B1] Aparicio V. A. Ortega F. B. Carbonell-Baeza A. Cuevas A. M. Delgado-Fernández M. Jonatan R. (2013). Anxiety, depression and fibromyalgia pain and severity. *Psicol. Conductual* 21 381–392.

[B2] Apkarian A. V. Bushnell M. C. Treede R.-D. Zubieta J.-K. (2005). Human brain mechanisms of pain perception and regulation in health and disease. *Eur. J. Pain* 9 463–463. 10.1016/j.ejpain.2004.11.001 15979027

[B3] Atlas L. Y. Wager T. D. (2012). How expectations shape pain. *Neurosci. Lett.* 520 140–148. 10.1016/j.neulet.2012.03.039 22465136

[B4] Babiloni C. Arendt-Nielsen L. Pascual-Marqui R. D. Rossini P. M. Brancucci A. Del Percio C. (2004). Cortical sensorimotor interactions during the expectancy of a Go/No-Go task: Effects of painful stimuli. *Behav. Neurosci.* 118 925–935. 10.1037/0735-7044.118.5.925 15506875

[B5] Bartley E. J. Rhudy J. L. Williams A. E. (2009). Experimental assessment of affective processing in fibromyalgia. *J. Pain* 10 1151–1160. 10.1016/j.jpain.2009.04.008 19632160

[B6] Beck A. T. Ward C. H. Mendelson M. Mock J. Erbaugh J. (1961). An inventory for measuring depression. *Arch. Gen. Psychiatry* 4 561–571. 13688369 10.1001/archpsyc.1961.01710120031004

[B7] Bell T. Trost Z. Buelow M. T. Clay O. Younger J. Moore D. (2018). Meta-analysis of cognitive performance in fibromyalgia. *J. Clin. Exp. Neuropsychol.* 40 698–714. 10.1080/13803395.2017.1422699 29388512 PMC6151134

[B8] Beydoun A. Morrow T. J. Shen J. F. Casey K. L. (1993). Variability of laser-evoked potentials: Attention, arousal and lateralized differences. *Electroencephalogr. Clin. Neurophysiol. Evoked Potentials* 88 173–181. 10.1016/0168-5597(93)90002-7 7684966

[B9] Boyle Y. El-Deredy W. Montes E. M. Bentley D. E. Jones A. K. P. (2008). Selective modulation of nociceptive processing due to noise distraction. *Pain* 138 630–640. 10.1016/j.pain.2008.02.020 18423872

[B10] Broadbent P. Liossi C. Schoth D. E. (2021). Attentional bias to somatosensory stimuli in chronic pain patients: A systematic review and meta-analysis. *Pain* 162 332–352. 10.1097/j.pain.0000000000002040 32833792

[B11] Brown C. (2017). “Anticipatory brain responses and expectancy effects on pain: Theory, research findings and functional networks,” in *Neuroimaging of pain*, (Cham: Springer International Publishing), 123–152. 10.1007/978-3-319-48046-6_6

[B12] Brown C. A. El-Deredy W. Jones A. K. P. (2014). When the brain expects pain: Common neural responses to pain anticipation are related to clinical pain and distress in fibromyalgia and osteoarthritis. *Eur. J. Neurosci.* 39 663–672. 10.1111/ejn.12420 24219587

[B13] Brown C. A. Seymour B. Boyle Y. El-Deredy W. Jones A. K. P. (2008). Modulation of pain ratings by expectation and uncertainty: Behavioral characteristics and anticipatory neural correlates. *Pain* 135 240–250. 10.1016/j.pain.2007.05.022 17614199

[B14] Brunia C. H. M. Hackley S. A. van Boxtel G. J. M. Kotani Y. Ohgami Y. (2011). Waiting to perceive: Reward or punishment? *Clin. Neurophysiol.* 122 858–868. 10.1016/j.clinph.2010.12.039 21215692

[B15] Brunia C. H. van Boxtel G. J. (2001). Wait and see. *Int. J. Psychophysiol.* 43 59–75. 10.1016/S0167-8760(01)00179-9 11742685

[B16] Burgmer M. Petzke F. Giesecke T. Gaubitz M. Heuft G. Pfleiderer B. (2011). Cerebral activation and catastrophizing during pain anticipation in patients with fibromyalgia. *Psychosom. Med.* 73 751–759. 10.1097/PSY.0b013e318236588a 22048836

[B17] Carretié L. Martín-Loeches M. Hinojosa J. A. Mercado F. (2001a). Emotion and attention interaction studied through event-related potentials. *J. Cogn. Neurosci.* 13 1109–1128. 10.1162/089892901753294400 11784449

[B18] Carretié L. Mercado F. Tapia M. (2001b). Emotion, attention, and the ‘ negativity bias ’, studied through event-related potentials. *Int. J. Psychophysiol.* 41 75–85. 11239699 10.1016/s0167-8760(00)00195-1

[B19] Carretié L. Tapia M. Mercado F. Albert J. López-Martín S. De La Serna J. M. (2004). Voltage-based versus factor score-based source localization analyses of electrophysiological brain activity: A comparison. *Brain Topogr.* 17 109–115. 10.1007/s10548-004-1008-1 15754876

[B20] Cattell R. (1966). The Scree Test for the number of factors. *Multivar. Behav. Res.* 1 116–141. 10.1207/s15327906mbr0102 26828106

[B21] Chapman S. L. Byas-Smith M. G. Reed B. A. (2002). Effects of intermediate- and long-term use of opioids on cognition in patients with chronic pain. *Clin. J. Pain* 18 S83–S90. 10.1097/00002508-200207001-00010 12479258

[B22] Cook D. B. Lange G. Ciccone D. S. Wen-Ching L. Steffener J. Natelson B. H. (2004). Functional Imaging of Pain in Patients with Primary Fibromyalgia. *J. Rheumatoly* 31 364–378. 14760810

[B23] Crombez G. Eccleston C. Van den Broeck A. Goubert L. Van Houdenhove B. (2004). Hypervigilance to Pain in Fibromyalgia. *Clin. J. Pain* 20 98–102. 10.1097/00002508-200403000-00006 14770049

[B24] Crombez G. Van Ryckeghem D. M. L. Eccleston C. Van Damme S. (2013). Attentional bias to pain-related information: A meta-analysis. *Pain* 154 497–510. 10.1016/j.pain.2012.11.013 23333054

[B25] Cui R. Egkher A. Huter D. Lang W. Lindinger G. Deecke L. (2000). High resolution spatiotemporal analysis of the contingent negative variation in simple or complex motor tasks and a non-motor task. *Clin. Neurophysiol.* 111 1847–1859. 10.1016/S1388-2457(00)00388-6 11018502

[B26] de Souza J. B. Potvin S. Goffaux P. Charest J. Marchand S. (2009). The deficit of pain inhibition in fibromyalgia is more pronounced in patients with comorbid depressive symptoms. *Clin. J. Pain* 25 123–127. 10.1097/AJP.0b013e318183cfa4 19333157

[B27] de Tommaso M. Federici A. Santostasi R. Calabrese R. Vecchio E. Lapadula G. (2011). Laser-evoked potentials habituation in fibromyalgia. *J. Pain* 12 116–124. 10.1016/j.jpain.2010.06.004 20685171

[B28] de Tommaso M. Ricci K. Libro G. Vecchio E. Delussi M. Montemurno A. (2017). Pain processing and vegetative dysfunction in fibromyalgia: A study by sympathetic skin response and laser evoked potentials. *Pain Res. Treat.* 2017 1–12. 10.1155/2017/9747148 29093972 PMC5637844

[B29] Dien J. (2010). The ERP PCA Toolkit: An open source program for advanced statistical analysis of event-related potential data. *J. Neurosci. Methods* 187 138–145. 10.1016/j.jneumeth.2009.12.009 20035787

[B30] Dien J. (2012). Applying principal components analysis to event-related potentials: A Tutorial. *Dev. Neuropsychol.* 37 497–517. 10.1080/87565641.2012.697503 22889342

[B31] Dien J. Santuzzi A. M. (2005). Application of repeated measures ANOVA to high-density ERP datasets: A review and tutorial. *Event Relat. Potentials A Methods Handb.* 4 57–82. 10.5061/dryad.30dn3

[B32] Dowman R. (2004). Electrophysiological indices of orienting attention toward pain. *Psychophysiology* 41 749–761. 10.1111/j.1469-8986.2004.00207.x 15318881

[B33] Dowman R. (2007). Neural mechanisms of detecting and orienting attention toward unattended threatening somatosensory targets. I. Intermodal effects. *Psychophysiology* 44 407–419. 10.1111/j.1469-8986.2007.00508.x 17371498

[B34] Dowman R. (2011). The role of somatic threat feature detectors in the attentional bias toward pain: Effects of spatial attention. *Psychophysiology* 48 397–409. 10.1111/j.1469-8986.2010.01068.x 20636292

[B35] Duschek S. Werner N. S. Limbert N. Winkelmann A. Montoya P. (2014). Attentional bias toward negative information in patients with fibromyalgia syndrome. *Pain Med.* 15 603–612. 10.1111/pme.12360 24447855

[B36] Ellingson L. D. Stegner A. J. Schwabacher I. J. Lindheimer J. B. Cook D. B. (2018). Catastrophizing interferes with cognitive modulation of pain in women with fibromyalgia. *Pain Med.* 19 2408–2422. 10.1093/pm/pny008 29474665 PMC6659027

[B37] Fairhurst M. Wiech K. Dunckley P. Tracey I. (2007). Anticipatory brainstem activity predicts neural processing of pain in humans. *Pain* 128 101–110. 10.1016/j.pain.2006.09.001 17070996

[B38] Fernandes-Magalhaes R. Ferrera D. Peláez I. Martín-Buro M. C. Carpio A. De Lahoz M. E. (2022). Neural correlates of the attentional bias towards pain-related faces in fibromyalgia patients: An ERP study using a dot-probe task. *Neuropsychologia* 166:108141. 10.1016/j.neuropsychologia.2021.108141 34995568

[B39] Ferrera D. Mercado F. Peláez I. Martínez-Iñigo D. Fernandes-Magalhaes R. Barjola P. (2021). Fear of pain moderates the relationship between self-reported fatigue and methionine allele of catechol-O-methyltransferase gene in patients with fibromyalgia. *PLoS One* 16:e0250547. 10.1371/journal.pone.0250547 33909692 PMC8081450

[B40] Fields H. L. (2018). How expectations influence pain. *Pain* 159 S3–S10. 10.1097/j.pain.0000000000001272 30113941

[B41] García-Larrea L. Peyron R. Laurent B. Mauguière F. (1997). Association and dissociation between laser-evoked potentials and pain perception. *Neuroreport* 8 3785–3789. 10.1097/00001756-199712010-00026 9427371

[B42] Geisser M. E. Casey K. L. Brucksch C. B. Ribbens C. M. Appleton B. B. Crofford L. J. (2003). Perception of noxious and innocuous heat stimulation among healthy women and women with fibromyalgia: Association with mood, somatic focus, and catastrophizing. *Pain* 102 243–250. 10.1016/S0304-3959(02)00417-7 12670665

[B43] Gibson S. Littlejohn G. O. Gorman M. Helme R. Granges G. (1994). Altered heat pain thresholds and cerebral event-related potentials following painful CO2 laser stimulation in subjects with fibromyalgia syndrome. *Pain* 58 185–193. 10.1016/0304-3959(94)90198-8 7816486

[B44] Giesecke T. Gracely R. H. Williams D. A. Geisser M. E. Petzke F. W. Clauw D. J. (2005). The relationship between depression, clinical pain, and experimental pain in a chronic pain cohort. *Arthritis Rheum.* 52 1577–1584. 10.1002/art.21008 15880832

[B45] Goffaux P. de Souza J. B. Potvin S. Marchand S. (2009). Pain relief through expectation supersedes descending inhibitory deficits in fibromyalgia patients. *Pain* 145 18–23. 10.1016/j.pain.2009.02.008 19524367

[B46] Gómez C. Marco J. Grau C. (2003). Preparatory visuo-motor cortical network of the contingent negative variation estimated by current density. *Neuroimage* 20 216–224. 10.1016/S1053-8119(03)00295-7 14527582

[B47] González-Roldán A. M. Bomba I. C. Diesch E. Montoya P. Flor H. Kamping S. (2016). Controllability and hippocampal activation during pain expectation in fibromyalgia syndrome. *Biol. Psychol.* 121 39–48. 10.1016/j.biopsycho.2016.09.007 27678310

[B48] González-Roldán A. M. Muñoz M. A. Cifre I. Sitges C. Montoya P. (2013). Altered psychophysiological responses to the view of others’. Pain and anger faces in fibromyalgia patients. *J. Pain* 14 709–719. 10.1016/j.jpain.2013.01.775 23623172

[B49] Gracely R. H. Petzke F. Wolf J. M. Clauw D. J. (2002). Functional magnetic resonance imaging evidence of augmented pain processing in fibromyalgia. *Arthritis Rheum.* 46 1333–1343. 10.1002/art.10225 12115241

[B50] Granovsky Y. Granot M. Nir R.-R. Yarnitsky D. (2008). Objective correlate of subjective pain perception by contact heat-evoked potentials. *J. Pain* 9 53–63. 10.1016/j.jpain.2007.08.010 17988951

[B51] Gratton G. Coles M. G. Donchin E. (1983). A new method for off-line removal of ocular artifact. *Electroencephalogr. Clin. Neurophysiol.* 55 468–484. 10.1016/0013-4694(83)90135-9 6187540

[B52] Gupta R. S. Kujawa A. Vago D. R. (2019). The neural chronometry of threat-related attentional bias?: Event-related potential (ERP) evidence for early and late stages of selective attentional processing. *Int. J. Psychophysiol.* 146 20–42. 10.1016/j.ijpsycho.2019.08.006 31605728 PMC6905495

[B53] Hauck M. Lorenz J. Zimmermann R. Debener S. Scharein E. Engel A. K. (2007). Duration of the cue-to-pain delay increases pain intensity: A combined EEG and MEG study. *Exp. Brain Res.* 180 205–215. 10.1007/s00221-007-0863-x 17287993

[B54] Henderson L. A. Di Pietro F. Youssef A. M. Lee S. Tam S. Akhter R. (2020). Effect of expectation on pain processing: A psychophysics and functional MRI analysis. *Front. Neurosci.* 14:6. 10.3389/fnins.2020.00006 32082106 PMC7004959

[B55] Hird E. J. Jones A. K. P. Talmi D. El-Deredy W. (2018). A comparison between the neural correlates of laser and electric pain stimulation and their modulation by expectation. *J. Neurosci. Methods* 293 117–127. 10.1016/j.jneumeth.2017.09.011 28935423

[B56] Huang Y. Shang Q. Dai S. Ma Q. (2017). Dread of uncertain pain: An event-related potential study. *PLoS One* 12:e0182489. 10.1371/journal.pone.0182489 28832607 PMC5568389

[B57] Iannetti G. D. Hughes N. P. Lee M. C. Mouraux A. (2008). Determinants of laser-evoked EEG responses: Pain perception or stimulus saliency? *J. Neurophysiol.* 100 815–828. 10.1152/jn.00097.2008 18525021 PMC2525705

[B58] Iannetti G. D. Zambreanu L. Cruccu G. Tracey I. (2005). Operculoinsular cortex encodes pain intensity at the earliest stages of cortical processing as indicated by amplitude of laser-evoked potentials in humans. *Neuroscience* 131 199–208. 10.1016/j.neuroscience.2004.10.035 15680703

[B59] Ioachim G. Warren H. J. M. Powers J. M. Staud R. Pukall C. F. Stroman P. W. (2022). Altered pain in the brainstem and spinal cord of fibromyalgia patients during the anticipation and experience of experimental pain. *Front. Neurol.* 13:862976. 10.3389/fneur.2022.862976 35599729 PMC9120571

[B60] Jenewein J. Moergeli H. Sprott H. Honegger D. Brunner L. Ettlin D. (2013). Fear-learning deficits in subjects with fibromyalgia syndrome? *Eur. J. Pain* 17 1374–1384. 10.1002/j.1532-2149.2013.00300.x 23468076 PMC3929307

[B61] Jennings J. R. van der Molen M. W. (2005). Preparation for speeded action as a psychophysiological concept. *Psychol. Bull.* 131 434–459. 10.1037/0033-2909.131.3.434 15869340

[B62] Jepma M. Koban L. van Doorn J. Jones M. Wager T. D. (2018). Behavioural and neural evidence for self-reinforcing expectancy effects on pain. *Nat. Hum. Behav.* 2 838–855. 10.1038/s41562-018-0455-8 31558818 PMC6768437

[B63] Johnston N. E. Atlas L. Y. Wager T. D. (2012). Opposing effects of expectancy and somatic focus on pain. *PLoS One* 7:e38854. 10.1371/journal.pone.0038854 22723896 PMC3378588

[B64] Kaji R. Ikeda A. Ikeda T. Kubori T. Mezaki T. Kohara N. (1995). Physiological study of cervical dystonia. Task-specific abnormality in contingent negative variation. *Brain* 118 511–522. 10.1093/brain/118.2.511 7735891

[B65] Kakigi R. Inui K. Tran D. T. Qiu Y. Wang X. Watanabe S. (2004). Human brain processing and central mechanisms of pain as observed by electro- and magneto-encephalography. *J. Chin. Med. Assoc.* 67 377–386. 15553795

[B66] Kamping S. Bomba I. C. Kanske P. Diesch E. Flor H. (2013). Deficient modulation of pain by a positive emotional context in fibromyalgia patients. *Pain* 154 1846–1855. 10.1016/j.pain.2013.06.003 23752177

[B67] Keltner J. R. Furst A. Fan C. Redfern R. Inglis B. Fields H. L. (2006). Isolating the modulatory effect of expectation on pain transmission: A functional magnetic resonance imaging study. *J. Neurosci.* 26 4437–4443. 10.1523/JNEUROSCI.4463-05.2006 16624963 PMC6674009

[B68] Kerns R. D. Turk D. C. Rudy T. E. (1985). The West Haven-Yale Multidimensional Pain Inventory (WHYMPI). *Pain* 23 345–356. 10.1016/0304-3959(85)90004-1 4088697

[B69] Knudsen L. Petersen G. L. Nørskov K. N. Vase L. Finnerup N. Jensen T. S. (2011). Review of neuroimaging studies related to pain modulation. *Scand. J. Pain* 2 108–120. 10.1016/j.sjpain.2011.05.005 29913745

[B70] Kong J. Jensen K. Loiotile R. Cheetham A. Wey H. Y. Tan Y. (2013). Functional connectivity of the frontoparietal network predicts cognitive modulation of pain. *Pain* 154 459–467. 10.1016/j.pain.2012.12.004 23352757 PMC3725961

[B71] Koyama T. McHaffie J. G. Laurienti P. J. Coghill R. C. (2005). The subjective experience of pain: Where expectations become reality. *Proc. Natl. Acad. Sci. U.S.A.* 102 12950–12955. 10.1073/pnas.0408576102 16150703 PMC1200254

[B72] Lai C. Ciacchella C. Pellicano G. R. Altavilla D. Sambucini D. Paolucci T. (2021). Different electrophysiological responses to pain-related visual stimuli between fibromyalgia and chronic low back pain women: A pilot case-control study. *Chronic Stress* 5:24705470211046881. 10.1177/24705470211046881 34988344 PMC8723168

[B73] Lee Y. C. Nassikas N. J. Clauw D. J. (2011). The role of the central nervous system in the generation and maintenance of chronic pain in rheumatoid arthritis, osteoarthritis and fibromyalgia. *Arthritis Res. Ther.* 13:211. 10.1186/ar3306 21542893 PMC3132050

[B74] Legrain V. Mancini F. Sambo C. F. Torta D. M. Ronga I. Valentini E. (2012). Cognitive aspects of nociception and pain. Bridging neurophysiology with cognitive psychology. *Neurophysiol. Clin.* 42 325–336. 10.1016/j.neucli.2012.06.003 23040703

[B75] Loggia M. L. Berna C. Kim J. Cahalan C. M. Gollub R. L. Wasan A. D. (2014). Disrupted brain circuitry for pain-related reward/punishment in fibromyalgia. *Arthritis Rheumatol.* 66 203–212. 10.1002/art.38191 24449585 PMC4516215

[B76] Lorenz J. (1998). Hyperalgesia or hypervigilance? An evoked potential approach to the study of fibromyalgia syndrome. *Z. Rheumatol.* 57 Suppl 2 19–22. 10025076 10.1007/s003930050228

[B77] Lorenz J. García-Larrea L. (2003). Contribution of attentional and cognitive factors to laser evoked brain potentials. *Neurophysiol. Clin.* 33 293–301. 10.1016/j.neucli.2003.10.004 14678843

[B78] Lorenz J. Grasedyck K. Bromm B. (1996). Middle and long latency somatosensory evoked potentials after painful laser stimulation in patients with fibromyalgia syndrome. *Electroencephalogr. Clin. Neurophysiol. Potentials Sect.* 100 165–168. 10.1016/0013-4694(95)00259-6 8617155

[B79] Lorenz J. Hauck M. Paur R. C. Nakamura Y. Zimmermann R. Bromm B. (2005). Cortical correlates of false expectations during pain intensity judgments–A possible manifestation of placebo/nocebo cognitions. *Brain Behav. Immun.* 19 283–295. 10.1016/j.bbi.2005.03.010 15890494

[B80] Luck S. J. (2014). “Overview of common ERP components,” in *In an introduction to the event-related potential technique*, (Cambridge, MA: MIT Press), 71–117.

[B81] McGowan N. Sharpe L. Refshauge K. Nicholas M. K. (2009). The effect of attentional re-training and threat expectancy in response to acute pain. *Pain* 142 101–107. 10.1016/j.pain.2008.12.009 19201093

[B82] Mercado F. Ferrera D. Fernandes-Magalhaes R. Peláez I. Barjola P. (2022). Altered subprocesses of working memory in patients with fibromyalgia: An event-related potential study using N -back task. *Pain Med.* 23 475–487. 10.1093/pm/pnab190 34145889

[B83] Meulders A. Jans A. Vlaeyen J. W. S. (2015). Differences in pain-related fear acquisition and generalization. *Pain* 156 108–122. 10.1016/j.pain.0000000000000016 25599307

[B84] Miyazaki M. Shibasaki H. Kanda M. Xu X. Shindo K. Honda M. (1994). Generator mechanism of pain-related evoked potentials following CO2 laser stimulation of the hand: Scalp topography and effect of predictive warning signal. *J. Clin. Neurophysiol.* 11 242–254. 10.1097/00004691-199403000-00010 8051309

[B85] Mohs R. Mease P. Arnold L. M. Wang F. Ahl J. Gaynor P. J. (2012). The effect of duloxetine treatment on cognition in patients with fibromyalgia. *Psychosom. Med.* 74 628–634. 10.1097/PSY.0b013e31825b9855 22753629

[B86] Moseley G. L. Arntz A. (2007). The context of a noxious stimulus affects the pain it evokes. *Pain* 133 64–71. 10.1016/j.pain.2007.03.002 17449180

[B87] Murga I. Guillen V. Lafuente J.-V. (2017). Cambios en la resonancia magnética cerebral asociados al síndrome de fibromialgia. *Med. Clin (Barc).* 148 511–516. 10.1016/j.medcli.2017.01.034 28450073

[B88] Ohara S. Crone N. E. Weiss N. Treede R. D. Lenz F. A. (2004). Amplitudes of laser evoked potential recorded from primary somatosensory, parasylvian and medial frontal cortex are graded with stimulus intensity. *Pain* 110 318–328. 10.1016/j.pain.2004.04.009 15275782

[B89] Palermo S. Benedetti F. Costa T. Amanzio M. (2015). Pain anticipation: An activation likelihood estimation meta-analysis of brain imaging studies. *Hum. Brain Mapp.* 36 1648–1661. 10.1002/hbm.22727 25529840 PMC6869158

[B90] Peláez I. Ferrera D. Barjola P. Fernandes R. Mercado F. (2019). Subliminal emotional pictures are capable of modulating early cerebral responses to pain in fibromyalgia. *PLoS One* 14:e0217909. 10.1371/journal.pone.0217909 31166997 PMC6550399

[B91] Peláez I. Martínez-Iñigo D. Barjola P. Cardoso S. Mercado F. (2016). Decreased pain perception by unconscious emotional pictures. *Front. Psychol.* 7:1636. 10.3389/fpsyg.2016.01636 27818642 PMC5073127

[B92] Peters M. L. Vlaeyen J. W. S. van Drunen C. (2000). Do fibromyalgia patients display hypervigilance for innocuous somatosensory stimuli? Application of a body scanning reaction time paradigm. *Pain* 86 283–292. 10.1016/S0304-3959(00)00259-1 10812258

[B93] Pidal-Miranda M. González-Villar A. J. Carrillo-de-la-Peña M. T. (2019). Pain expressions and inhibitory control in patients with fibromyalgia: Behavioral and neural correlates. *Front. Behav. Neurosci.* 12:323. 10.3389/fnbeh.2018.00323 30670955 PMC6332144

[B94] Plaghki L. Mouraux A. (2005). EEG and laser stimulation as tools for pain research. *Curr. Opin. Investig. Drugs* 6 58–64. 15675604

[B95] Ploghaus A. Becerra L. Borras C. Borsook D. (2003). Neural circuitry underlying pain modulation: Expectation, hypnosis, placebo. *Trends Cogn. Sci.* 7 197–200. 10.1016/S1364-6613(03)00061-5 12757820

[B96] Rainville P. (2002). Brain mechanisms of pain affect and pain modulation. *Curr. Opin. Neurobiol.* 12 195–204. 10.1016/S0959-4388(02)00313-6 12015237

[B97] Reyes del Paso G. A. Pulgar Á Duschek S. Garrido S. (2012). Cognitive impairment in fibromyalgia syndrome: The impact of cardiovascular regulation, pain, emotional disorders and medication. *Eur. J. Pain* 16 421–429. 10.1002/j.1532-2149.2011.00032.x 22337559

[B98] Richard J. Y. Hurley R. A. Taber K. H. (2019). Fibromyalgia: Centralized Pain Processing and Neuroimaging. *J. Neuropsychiatry Clin. Neurosci.* 31 A6–A187. 10.1176/appi.neuropsych.19050107 31322995

[B99] Rizzo P. A. Pierelli F. Pozzessere G. Fattapposta F. Sanarelli L. Morocutti C. (1985). Pain, anxiety, and contingent negative variation: A clinical and pharmacological study. *Biol. Psychiatry* 20 1297–1302. 10.1016/0006-3223(85)90114-3 4063419

[B100] Rohrbaugh J. W. Gaillard A. W. K. (1983). “Sensory and motor aspects of the contingent negative variation,” in *Advances in psychology*, eds Gaillard A. W. K. Ritter W. (Amsterdam: Elsevier), 269–310. 10.1016/S0166-4115(08)62044-0

[B101] Rosahl S. K. Knight R. T. (1995). Role of prefrontal cortex in generation of the contingent negative variation. *Cereb. Cortex* 5 123–134. 10.1093/cercor/5.2.123 7620289

[B102] Sandström A. Ellerbrock I. Tour J. Kadetoff D. Jensen K. B. Kosek E. (2020). Neural correlates of conditioned pain responses in fibromyalgia subjects indicate preferential formation of new pain associations rather than extinction of irrelevant ones. *Pain* 161 2079–2088. 10.1097/j.pain.0000000000001907 32379218 PMC7431138

[B103] Sawamoto N. Honda M. Okada T. Hanakawa T. Kanda M. Fukuyama H. (2000). Expectation of pain enhances responses to nonpainful somatosensory stimulation in the anterior cingulate cortex and parietal operculum/posterior insula: An event-related functional magnetic resonance imaging study. *J. Neurosci.* 20 7438–7445. 10.1523/jneurosci.20-19-07438.2000 11007903 PMC6772793

[B104] Schneider C. Palomba D. Flor H. (2004). Pavlovian conditioning of muscular responses in chronic pain patients: Central and peripheral correlates. *Pain* 112 239–247. 10.1016/j.pain.2004.08.025 15561378

[B105] Schoth D. E. Nunes V. D. Liossi C. (2012). Attentional bias towards pain-related information in chronic pain; a meta-analysis of visual-probe investigations. *Clin. Psychol. Rev.* 32 13–25. 10.1016/j.cpr.2011.09.004 22100743

[B106] Schweinhardt P. Kalk N. Wartolowska K. Chessell I. Wordsworth P. Tracey I. (2008). Investigation into the neural correlates of emotional augmentation of clinical pain. *Neuroimage* 40 759–766. 10.1016/j.neuroimage.2007.12.016 18221890

[B107] Seidel E.-M. Pfabigan D. M. Hahn A. Sladky R. Grahl A. Paul K. (2015). Uncertainty during pain anticipation: The adaptive value of preparatory processes. *Hum. Brain Mapp.* 36 744–755. 10.1002/hbm.22661 25324216 PMC6869185

[B108] Shih Y.-W. Tsai H.-Y. Lin F.-S. Lin Y.-H. Chiang C.-Y. Lu Z.-L. (2019). Effects of positive and negative expectations on human pain perception engage separate but interrelated and dependently regulated cerebral mechanisms. *J. Neurosci.* 39 1261–1274. 10.1523/JNEUROSCI.2154-18.2018 30552181 PMC6381241

[B109] Sitges C. González-Roldán A. M. Duschek S. Montoya P. (2018). Emotional influences on cognitive processing in fibromyalgia patients with different depression levels: An event-related potential study. *Clin. J. Pain* 34 1106–1113. 10.1097/AJP.0000000000000637 29975206

[B110] Spielberger C. D. Gorsuch R. L. Lushene R. (1982). *Manual del Cuestionario de Ansiedad Estado/Rasgo (STAI).* Madrid: TEA Ediciones.

[B111] Stude P. Wischniewski C. Thümler P. Lehmenkühler A. Richter F. Wiemann M. (2003). Scalp-recorded contingent negative variation (CNV) increases during experimentally induced sustained ischemic pain in humans. *Neurosci. Lett.* 348 9–12. 10.1016/S0304-3940(03)00642-6 12893413

[B112] Todd J. van Ryckeghem D. M. L. Sharpe L. Crombez G. (2018). Attentional bias to pain-related information: A meta-analysis of dot-probe studies. *Health Psychol. Rev.* 12 419–436. 10.1080/17437199.2018.1521729 30205757

[B113] Treede R.-D. Lorenz J. Baumgärtner U. (2003). Clinical usefulness of laser-evoked potentials. *Neurophysiol. Clin. Neurophysiol.* 33 303–314. 10.1016/j.neucli.2003.10.009 14678844

[B114] Van Damme S. Crombez G. Eccleston C. (2002). Retarded disengagement from pain cues: The effects of pain catastrophizing and pain expectancy. *Pain* 100 111–118. 10.1016/S0304-3959(02)00290-7 12435464

[B115] Van Damme S. Crombez G. Eccleston C. Goubert L. (2004). Impaired disengagement from threatening cues of impending pain in a crossmodal cueing paradigm. *Eur. J. Pain* 8 227–236. 10.1016/j.ejpain.2003.08.005 15109973

[B116] Van Damme S. Legrain V. Vogt J. Crombez G. (2010). Keeping pain in mind: A motivational account of attention to pain. *Neurosci. Biobehav. Rev.* 34 204–213. 10.1016/j.neubiorev.2009.01.005 19896002

[B117] Van Damme S. Van Hulle L. Spence C. Devulder J. Brusselmans G. Crombez G. (2015). Hypervigilance for innocuous tactile stimuli in patients with fibromyalgia: An experimental approach. *Eur. J. Pain* 19 706–714. 10.1002/ejp.593 25252089

[B118] Van Ryckeghem D. M. L. Noel M. Sharpe L. Pincus T. Van Damme S. (2019). Cognitive biases in pain: An integrated functional–contextual framework. *Pain* 160 1489–1493. 10.1097/j.pain.0000000000001508 30913168

[B119] Van Ryckeghem D. Crombez G. (2015). General hypervigilance in fibromyalgia: One swallow does not make a summer. *Eur. J. Pain* 19 447–448. 10.1002/ejp.658 25691446

[B120] Vecchio E. Quitadamo S. G. Ricci K. Libro G. Delussi M. Lombardi R. (2022). Laser evoked potentials in fibromyalgia with peripheral small fiber involvement. *Clin. Neurophysiol.* 135 96–106. 10.1016/j.clinph.2022.01.001 35074721

[B121] Vlaeyen J. W. S. Morley S. Crombez G. (2016). The experimental analysis of the interruptive, interfering, and identity-distorting effects of chronic pain. *Behav. Res. Ther.* 86 23–34. 10.1016/j.brat.2016.08.016 27614948

[B122] Weiss T. Miltner W. H. R. Dillmann J. Miltner W. H. R. Weiss T. Miltner W. H. R. (2003). The influence of semantic priming on event-related potentials to painful laser-heat stimuli in migraine patients. *Neurosci. Lett.* 340 135–138. 10.1016/S0304-3940(03)00103-4 12668255

[B123] Wiech K. (2016). Deconstructing the sensation of pain: The influence of cognitive processes on pain perception. *Science* 354 584–587. 10.1126/science.aaf8934 27811269

[B124] Wiech K. Ploner M. Tracey I. (2008). Neurocognitive aspects of pain perception. *Trends Cogn. Sci.* 12 306–313. 10.1016/j.tics.2008.05.005 18606561

[B125] Wolfe F. Clauw D. J. Fitzcharles M.-A. Goldenberg D. L. Häuser W. Katz R. L. (2016). 2016 Revisions to the 2010/2011 fibromyalgia diagnostic criteria. *Semin. Arthritis Rheum.* 46 319–329. 10.1016/j.semarthrit.2016.08.012 27916278

[B126] Wolfe F. Smythe H. A. Yunus M. B. Bennett R. M. Bombardier C. Goldenberg D. L. (1990). The american college of rheumatology 1990 criteria for the classification of fibromyalgia. Report of the Multicenter Criteria Committee. *Arthritis Rheum.* 33 160–172. 10.1002/art.1780330203 2306288

[B127] Yiend J. Mathews A. (2001). Anxiety and attention to threatening pictures. *Q. J. Exp. Psychol. A* 54 665–681. 10.1080/713755991 11548029

[B128] Zhang L. Lu X. Bi Y. Hu L. (2019). Pavlov’s Pain: The effect of classical conditioning on pain perception and its clinical implications. *Curr. Pain Headache Rep.* 23:19. 10.1007/s11916-019-0766-0 30835004

